# Combination of epigallocatechin 3 gallate and curcumin improves d-galactose and normal-aging associated memory impairment in mice

**DOI:** 10.1038/s41598-023-39919-4

**Published:** 2023-08-04

**Authors:** Md. Ashrafur Rahman, Arif Anzum Shuvo, Md. Mehedi Hasan Apu, Monisha Rani Bhakta, Farzana Islam, Md. Atiqur Rahman, Md. Rabiul Islam, Hasan Mahmud Reza

**Affiliations:** 1 Department of Pharmaceutical Sciences, North South University, Bashundhara, Dhaka, 1229 Bangladesh; 2 Department of Pharmaceutical Sciences, Wilkes University, Wilkes Barre, PA 18766 USA; 3 Department of Pharmacy, University of Asia Pacific, 74/A Green Road, Farmgate, Dhaka, 1205 Bangladesh; 4 School of Pharmacy, BRAC University, 66 Mohakhali, Dhaka, 1212 Bangladesh

**Keywords:** Neuroscience, Neurology

## Abstract

Previously, we observed curcumin improves aging-associated memory impairment in d-galactose (D-gal) and normal-aged (NA) mice. Evidence showed that multiple agents can be used in managing aging-induced memory dysfunction, drawn by the contribution of several pathways. Curcumin and Epigallocatechin 3 gallate (EGCG) combination substantially reduced the oxidative stress that commonly mediates aging. This study examined the combined effect of EGCG and curcumin on memory improvement in two recognized models, D-gal and normal-aged (NA) mice. The co-administration of EGCG and curcumin significantly (p < 0.05) increased retention time detected by passive avoidance (PA) and freezing response determined in contextual fear conditioning (CFC) compared to the discrete administration of EGCG or curcumin. Biochemical studies revealed that the combination of EGCG and curcumin remarkably ameliorated the levels (p < 0.05) of glutathione, superoxide dismutase, catalase, advanced oxidation protein products, nitric oxide, and lipid peroxidation compared to the monotherapy of EGCG or curcumin in mice hippocampi. The behavioral and biochemical studies revealed that the combination of EGCG and curcumin showed better improvement in rescuing aging-associated memory disorders in mice. EGCG and curcumin combination could serve as a better choice in managing aging-related memory disorders.

## Introduction

Aging, a progressive physiological change or decline in biological function^1^, causes the deterioration of several organs, especially the brain^2^. Brain aging characterizes molecular and cellular modifications in the hippocampus, cortical density, and neurotransmitter systems^3^. These changes emerge in multiple ways, including oxidative stress-induced injury^4^. A disparity among antioxidant enzyme activities, species containing reactive oxygen (ROS) and reactive nitrogen (RNS), protein oxidation, lipid peroxidation, and mitochondrial DNA (mtDNA) damage evokes brain aging, resulting in memory impairment^5–7^. This dysfunction in learning and memory due to oxidative stress can be developed in d galactose (D-gal)-induced accelerated brain aging model that causes deficits in behaviors, neuro, and biochemistry similar to natural aging^8,9^. One study demonstrated that a moderate administration of D-gal can develop a safe aging model of rodent within a short time to screen anti-aging drugs for neurodegenerative disorders including memory impairment^8^. Studies showed that D-gal facilitates brain aging and accompanied cognitive dysfunctions by causing an impairment of adenosine triphosphate (ATP) production, abnormal redox equilibrium, the escalation of nicotinamide adenine dinucleotide phosphate (NADPH) oxidase, glycotoxins (advanced glycation end products, AGEs) and their receptors (RAGEs)^10^. These cause the uncontrolled production of ROS that stimulates oxidative reactions, such as mitochondrial dysfunction, inflammation, cellular apoptosis, and neuronal degeneration^11^. One study demonstrated that D-gal oxidizes into aldehydes and hydrogen peroxide (H_2_O_2_) by galactose oxidase^12^ and triggers aging-associated memory loss^13^ in several behavioral batteries^14^. Our former study showed that D-gal causes deterioration of retention and fear memory by inducing oxidative overload in mouse hippocampus^15^.

Evidence showed that natural compounds can be the rational choice for treating aging-related memory disorder owing to antioxidants, anti-inflammatory, anti-aging^16^, and safer therapeutic properties^17^. It was reported that Epigallocatechin 3 gallate (EGCG), a natural polyphenolic agent of green tea^18^, improved memory and learning process by modulating the amount of several oxidative markers, including superoxide dismutase (SOD), malondialdehyde (MDA), and nitric oxide^19^. One study showed that EGCG decreased acetylcholinesterase (AChE) activity by ameliorating antioxidants and ROS in dementia rat models^20^. It has also been evident that EGCG improves memory impairment in passive avoidance (PA)^21^ and contextual fear conditioning (CFC)^22^ in the aging studies. Similarly, another natural compound, curcumin improved retention time in PA and freezing response in CFC tasks in D-gal injected aging animal model^15^. These improvements in behavioral endophenotype appear due to the antioxidant, anti-inflammatory, and anti-senescence^23^ properties of curcumin, shown in a previous study^15^. Contrarily, multiple factors accelerate the aging process^24,25^ which involves diverse pathways and interactive networks of components at molecular and cellular levels^26^. Therefore, considering curcumin alone to treat memory dysfunction in our previous study^15^ certainly missed finding out more substantial protection from aging.

In vitro study showed that curcumin possesses synergistic effects on EGCG^27^. Contrary, limited study has been conducted to find out a suitable adjuvant candidate of EGCG in treating aging-induced memory impairment. This study examined the combined effect of EGCG and curcumin on two widely used animal models, Normal aging and D-gal-administered aging mice, adopting behavioral paradigms, PA and CFC. We also assessed the oxidative stress biomarkers, such as glutathione (GSH), superoxide dismutase (SOD), catalase (CAT), advanced oxidation of protein products (AOPP), nitric oxide (NO), and malondialdehyde (MDA) in the animal hippocampus. Overall, this study has examined the combined effect of EGCG, and curcumin for the very first time on aging-associated memory disorder by behavioral and biochemical tests in mice.

## Results

### Effect of EGCG + curcumin on retention time in passive avoidance task

**Figure 1:**
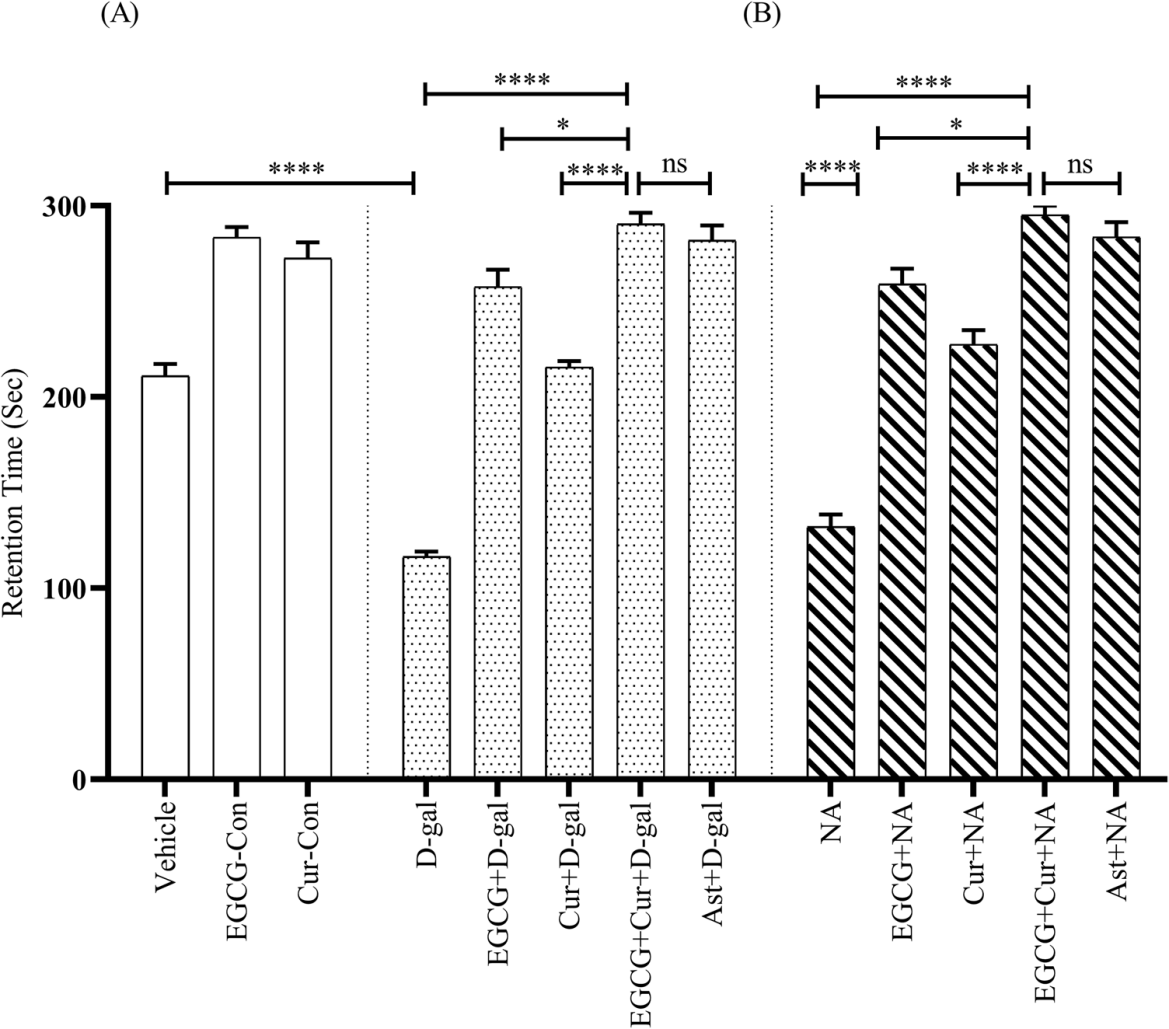
Effect of EGCG + curcumin on RT in D-gal (**A**) and NA (**B**) mice group after 24 h of training. The RT was calculated by performing PA tasks among young adult (vehicle, EGCG-Con, Cur-Con), drug induced aging (D-gal, EGCG + D-gal, Cur + D-gal, EGCG + Cur + D-gal, and Ast + D-gal), and nature induced aging (NA, EGCG + NA, Cur + NA, EGCG + Cur + NA, and Ast + NA) groups. RT was expressed in second. Data was presented as mean ± SEM, n = 8 each group; ****p < 0.0001, *ns* not significant.

A statistical comparison across the aging (Young, D-gal, and NA) and treatment (EGCG, Cur, EGCG + Cur, Ast, and no treatment), using two-way ANOVA followed by post hoc Tukey’s test, revealed a significant effect of RT after 24 h of training (Fig. 1A,B) on aging (F_2,91_ = 71.30, p < 0.0001), treatment (F_4,91_ = 230.75, p < 0.0001) and a significant interaction between aging and treatment (F_6,91_ = 5.32, p < 0.0001). The Vehicle, EGCG-Control (EGCG-Con), and Curcumin-Control (Cur-Con) mice exhibited retention times (RTs, mean ± standard error of the mean) of 210.93 ± 6.33 s, 283.5 ± 5.31 s, and 272.56 ± 8.32 s, respectively (Fig. 1A). The D-gal treated and NA mice groups showed a low level of RT value (116.43 ± 2.62 s, Fig. 1A; 132.12 ± 3.11 s, Fig. 1B, respectively), demonstrating a significant change in RT compared with the Vehicle (p < 0.0001), EGCG-Con (p < 0.0001), and Cur-Con (p < 0.0001), groups (Fig. 1A,B). Conversely, in contrast to the monotherapy of EGCG (257.56 ± 9.05 s) or curcumin (215.62 ± 3.19 s) in D-gal and EGCG (258.93 ± 8.21 s) or curcumin (227.50 ± 7.41 s) in NA, much greater prevention from the dropping trend of RT was seen in the combined treatment of EGCG and curcumin in D-gal (290.43 ± 5.98 s; F_1,14_ = 1.48, p < 0.05; F_1,14_ = 0.41, p < 0.001; respectively) and NA (295.12 ± 4.87 s; F_1,14_ = 1.62, p < 0.01; F_1,14_ = 1.58, p < 0.0001; respectively) groups mice, detected by One-Way ANOVA followed by post hoc Tukey’s test. The RT observed in the co-administered EGCG and curcumin was comparable to the RT detected in Ast + D-gal (281.87 ± 7.79 s; p > 0.05; Fig. 1A) and Ast + NA (283.62 ± 7.71 s; p > 0.05; Fig. 1B) mice groups.

A similar pattern was apparent after 48 h of training (Fig. S1A,B). Two-way ANOVA followed by post hoc Tukey’s test demonstrated a significant effect of aging (F_2,91_ = 69.16, p < 0.0001), treatment (F_4,91_ = 246.58, p < 0.0001) as well as a significant interaction between aging and treatment (F_6,91_ = 5.92, p < 0.0001). The combined EGCG and curcumin therapy demonstrated a significant difference in preventing the dropping trend of RT in D-gal (286.5 ± 6.37 s; Fig S1A) and NA (292.62 ± 7.37; Fig S1B) compared to the discrete administration of EGCG (F_1,14_ = 0.53, p < 0.05; Fig S1A) or curcumin (F_1,14_ = 2.51, p < 0.0001; Fig S1A) in D-gal and EGCG (F_1,14_ = 0.02, p < 0.05; Fig S1B) or curcumin (F_1,14_ = 0.14, p < 0.0001; Fig S1B) in NA, respectively, revealed by One-Way ANOVA followed by post hoc Tukey’s test. The RT found in co-administered EGCG and curcumin was equivalent to the RT seen in the Ast + D-gal (272.25 ± 7.80 s; p > 0.05; Fig S1A) and Ast + NA (279.87 ± 9.62 s; p > 0.05; Fig S1B) groups.

### Effect of EGCG + curcumin on freezing response in contextual fear conditioning task

#### Effect of EGCG + curcumin on freezing response in conditioning session

**Figure 2:**
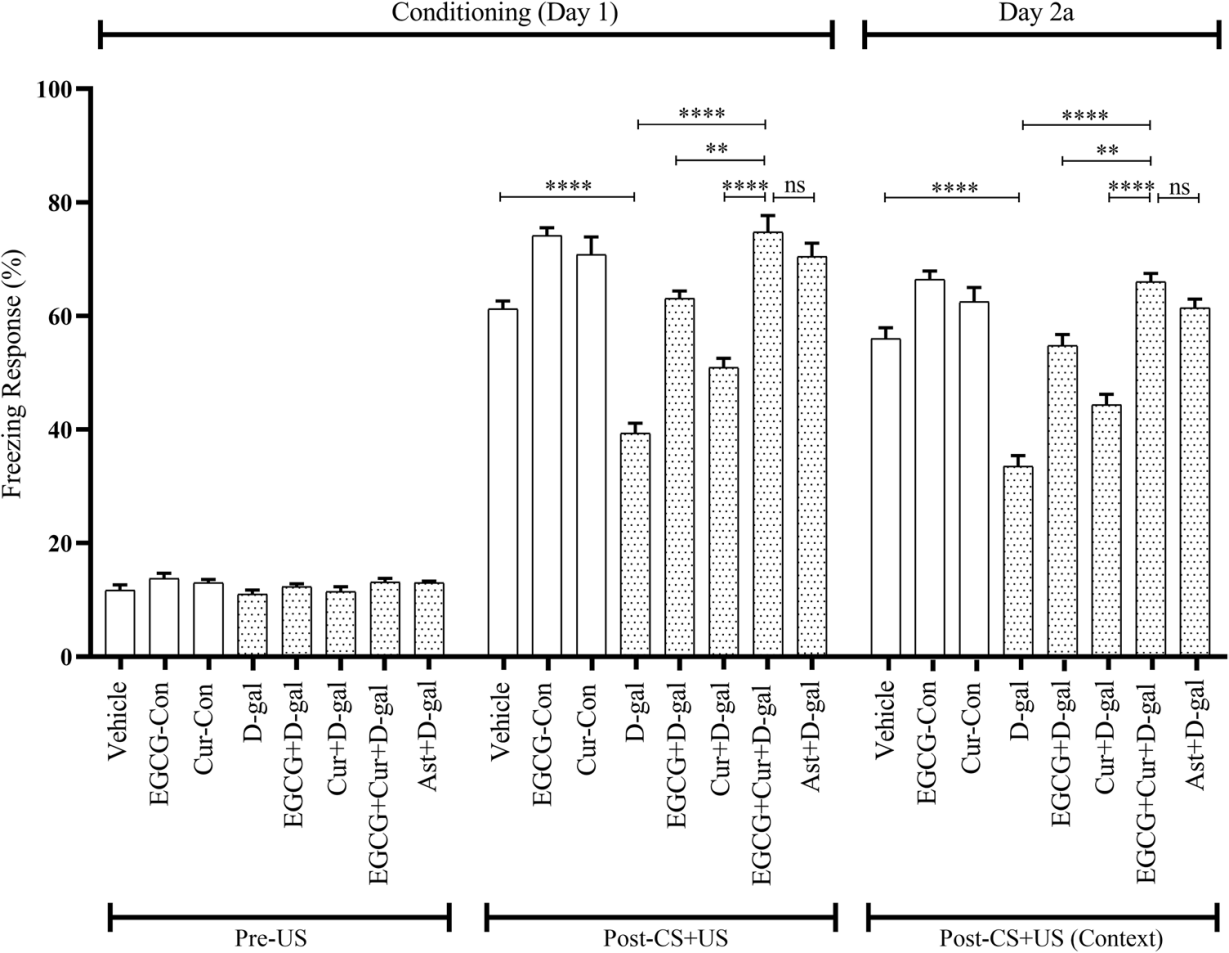
Effect of EGCG + Curcumin on the conditioning session (Day 1 of CFC) and context test (Day 2a) of D-gal mice group. The memory was assessed by analyzing the FR. The FR was expressed in percentage (%). Data was presented as mean ± SEM, n = 8 each group; **p < 0.01, ***p < 0.001, ****p < 0.0001, *ns* not significant.

**Figure 3:**
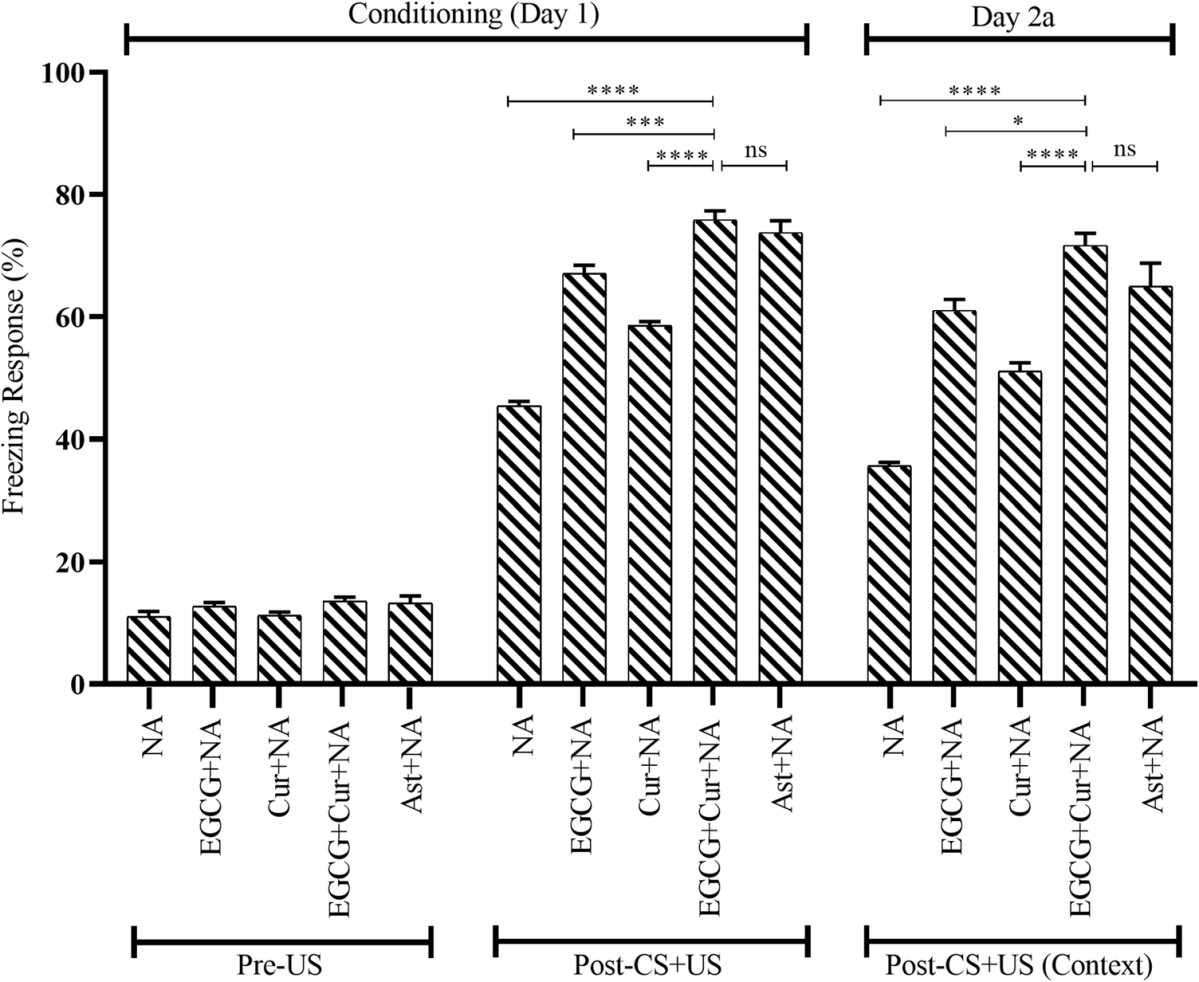
Effect of EGCG + Curcumin on the conditioning session (Day 1 of CFC) and context test (Day 2a) of NA mice group. The memory was assessed by analyzing the FR. The FR was expressed in percentage (%). Data was presented as mean ± SEM, n = 8 each group; **p < 0.01, ***p < 0.001, ****p < 0.0001, *ns* not significant.

To evaluate the tendency of hyperactivity to a fearful condition, the baseline activity in the first 2 min of the novel environment was detected in conditioning session on day 1 of CFC. This session measured the freezing response (FR) without the application of white noise (CS) and foot shock (US). The baseline activity among the Vehicle, EGCG-Con, Cur-Con, D-gal, NA, EGCG + D-gal, EGCG + NA, Cur + D-gal, Cur + NA, EGCG + Cur + D-gal, EGCG + Cur + NA, Ast + D-gal, Ast + NA groups (Figs. 2, 3) was almost identical.

A statistical comparison across the aging (Young, D-gal, and NA) and treatment (EGCG, Cur, EGCG + Cur, Ast, and no treatment), using a two-way ANOVA followed by post hoc Tukey’s test, detected a significant effect of FR of conditioning session (Figs. 2, 3) on aging (F_2,91_ = 96.29, p < 0.0001), treatment (F_4,91_ = 133.86, p < 0.0001) and a significant interaction between aging and treatment (F_6,91_ = 2.50, p < 0.05). In the presence of CS-US pairings at the last 6 min of conditioning session, a low FR was exhibited in the D-gal (39.37 ± 1.75%; Fig. 2) and NA (45.41 ± 0.75%; Fig. 3), denoting a statistically significant change compared to the Vehicle (61.25 ± 1.39%; p < 0.0001), EGCG-Con (74.16 ± 1.37%; p < 0.0001), and Cur-Con (70.83 ± 3.11%; p < 0.0001), respectively (Figs. 2, 3). Contrary, in comparison to the monotherapy of EGCG (63.12 ± 1.26%; Fig. 2) or curcumin (50.93 ± 1.60%; Fig. 2) in D-gal and EGCG (67.08 ± 1.32%; Fig. 3) or curcumin (58.54 ± 0.66%, Fig. 3) in NA, much higher prevention from the dropping trend of FR was detected by the combined EGCG and curcumin in D-gal (74.79 ± 2.88%; F_1,14_ = 3.96, p < 0.01; F_1,14_ = 2.14, p < 0.0001, respectively; Fig. 2) and NA (75.83 ± 1.47%; F_1,14_ = 0.32, p < 0.001; F_1,14_ = 8.17, p < 0.0001, respectively; Fig. 3) groups mice, demonstrated by One-Way ANOVA followed by post hoc Tukey’s test. The FR observed in the co-administered EGCG and curcumin was equivalent to the FR of Ast + D-gal (70.52 ± 2.30%; p > 0.05; Fig. 2) and Ast + NA (73.75 ± 1.93%; p > 0.05; Fig. 3) groups.

#### Effect of EGCG + curcumin on freezing response in context test

**Figure 4:**
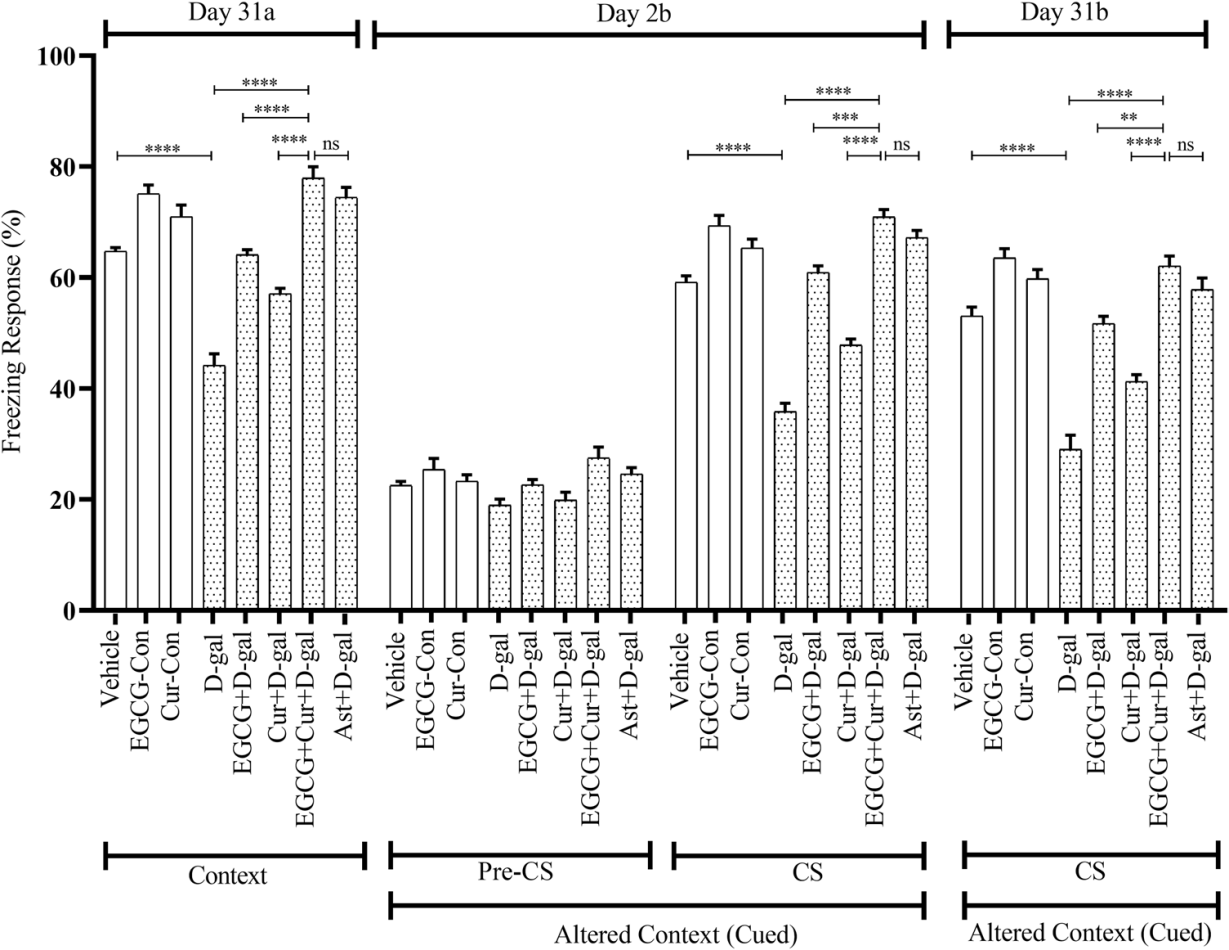
Effect of EGCG + Curcumin on the context (Day 31a) and cued fear memory (Day 2b and 31b) of D-gal mice group. The memory was assessed by analyzing the FR. The FR was expressed in percentage (%). Data was presented as mean ± SEM, n = 8 each group; *p < 0.05, **p < 0.01, ***p < 0.001, ****p < 0.0001, *ns* not significant.

**Figure 5:**
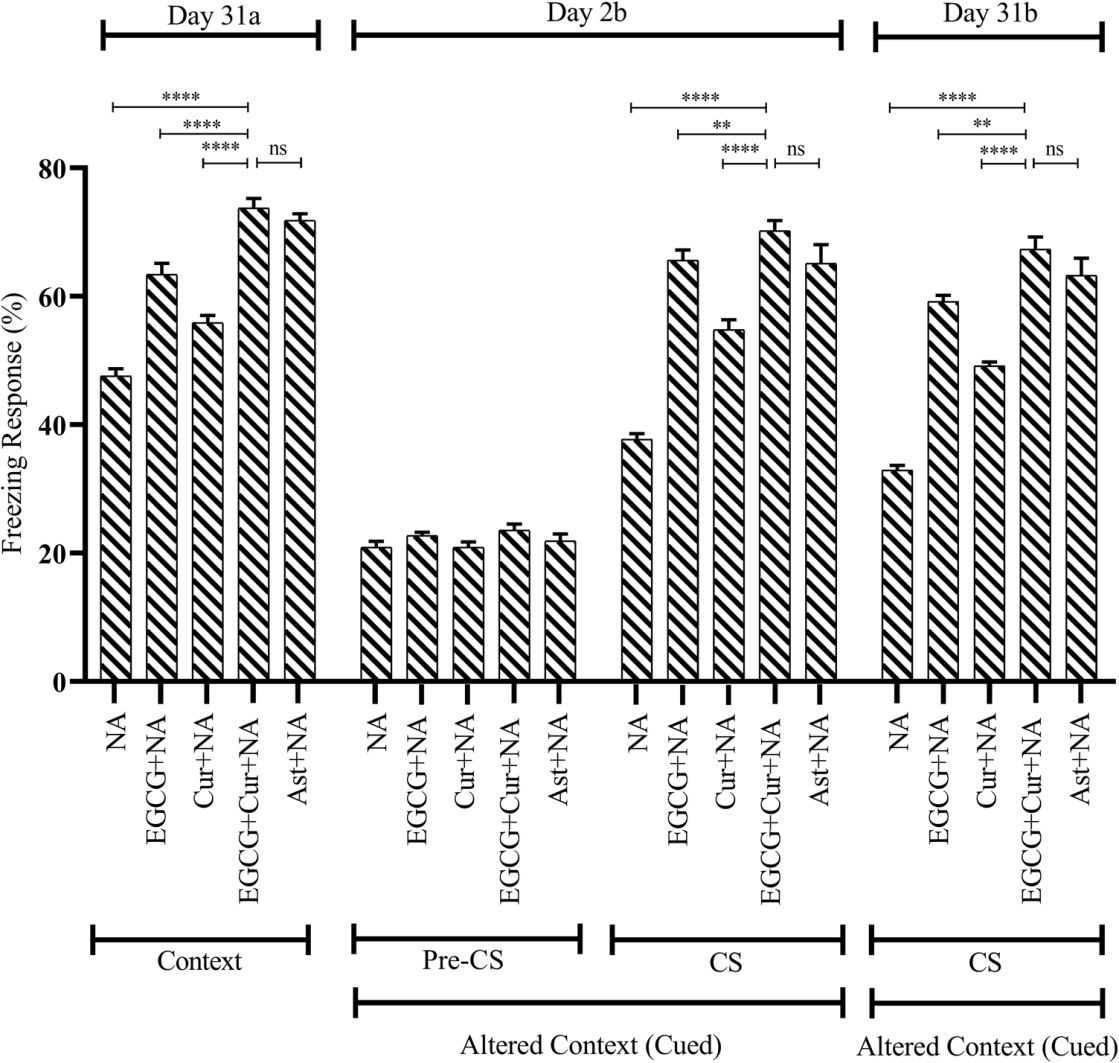
Effect of EGCG + Curcumin on the context (Day 31a) and cued fear memory (Day 2b and 31b) of NA mice group. The memory was assessed by analyzing the FR. The FR was expressed in percentage (%). Data was presented as mean ± SEM, n = 8 each group; *p < 0.05, **p < 0.01, ***p < 0.001, ****p < 0.0001, *ns* not significant.

A statistical comparison across the aging (Young, D-gal, and NA) and treatment (EGCG, Cur, EGCG + Cur, Ast, and no treatment), using a two-way ANOVA followed by post hoc Tukey’s test, demonstrated a significant effect of FR of context (after 24 h of conditioning) on aging on day 2a (F_2,91_ = 71.98, p < 0.0001; Figs. 2, 3) and 31a (F_2,91_ = 63, p < 0.0001; Figs. 4, 5), treatment on day 2a (F_4,91_ = 113.56, p < 0.0001) and 31a (F_4,91_ = 92.02, p < 0.0001) as well as a significant interaction between aging and treatment on day 2a (F_6,91_ = 2.35, p < 0.05), and 31a (F_6,91_ = 2.63, p < 0.05).

In the presence of CS-US pairings at the duration of the last 6 min on day 2a of context, contrasting to the monotherapy of EGCG (54.79 ± 2.25%; Fig. 2) or curcumin (44.37 ± 1.83%; Fig. 2) in D-gal and EGCG (61.04 ± 1.80%; Fig. 3) or curcumin (51.04 ± 1.40%; Fig. 3) in NA, there was a statistically significant difference of preventing the downward trend of FR in the combined administration of EGCG and curcumin in D-gal (66.04 ± 1.47%; F_1,14_ = 2.16, p < 0.01; F_1,14_ = 0.36, p < 0.0001, respectively; Fig. 2) and NA (71.66 ± 1.96%; F_1,14_ = 0.20, p < 0.05; F_1,14_ = 1.18, p < 0.0001, respectively; Fig. 3) groups (One-Way ANOVA followed by post hoc Tukey’s test). This FR distinguished in the combined EGCG and curcumin-treated mice was equivalent to the FR detected in the Ast + D-gal (61.45 ± 1.52%; p > 0.05; Fig. 2) and Ast + NA group mice (65 ± 3.74%; p > 0.05; Fig. 3).

A similar freezing response was observed on day 31a of the context (after 30 days of conditioning) test. In contrast to the discrete treatment of EGCG (64.09 ± 0.93%; Fig. 4) or curcumin (57.08 ± 0.97%; Fig. 4) in D-gal and EGCG (63.40 ± 1.73%; Fig. 5) or curcumin (55.90 ± 1.13%; Fig. 5) in NA, the co-administered treatment of EGCG and curcumin had a much greater protection from the dropping trend of FR in D-gal (77.91 ± 2.07%; F_1,14_ = 13.12, p < 0.0001; F_1,14_ = 12.19, p < 0.0001, respectively; Fig. 4) and NA (73.75 ± 1.49%; F_1,14_ = 0.05, p < 0.0001; F_1,14_ = 0.19, p < 0.0001, respectively; Fig. 5) mice (One-Way ANOVA followed by post hoc Tukey’s test). The FR observed in combined EGCG and curcumin groups were comparable to the FR observed in Ast + D-gal (74.44 ± 1.83%; p > 0.05, Fig. 4) and Ast + NA (71.80 ± 1.08%; p > 0.05, Fig. 5) groups.

#### Effect of EGCG + curcumin on FR in cued test

In the cued trial, the freezing response of mice investigated an altered context in a distinctly shaped chamber. In the presence of Pre-CS application during the first 3 min, the baseline activity among the Vehicle, EGCG-Con, Cur-Con, D-gal, NA, EGCG + D-gal, EGCG + NA, Cur + D-gal, Cur + NA, EGCG + Cur + D-gal, EGCG + Cur + NA, Ast + D-gal, Ast + NA groups (Figs. 4, 5) was approximately identical.

A statistical comparison across the aging (Young, D-gal, and NA) and treatment (EGCG, Cur, EGCG + Cur, Ast, and no treatment), using a two-way ANOVA followed by post hoc Tukey’s test, detected a significant effect of FR of cued (after performing day 2a and day 31a of context) on aging on day 2b (F_2,91_ = 82.23, p < 0.0001; Figs. 4, 5) and 31b (F_2,91_ = 89.87, p < 0.0001; Figs. 4, 5), treatment on day 2b (F_4,91_ = 145.36, p < 0.0001) and 31b (F_4,91_ = 122.17, p < 0.0001) and a significant interaction between aging and treatment on day 2b (F_6,91_ = 7.74, p < 0.0001) and 31b (F_6,91_ = 3.95, p = 0.001). On day 2b (After performing day 2a of context), in the presence of CS application during the last 3 min of the cued, a significantly low percent of FR was detected in D-gal (35.83 ± 1.50%; Fig. 4) and NA (37.70 ± 0.91%; Fig. 5) mice. In contrast to the monotherapy of EGCG (60.93 ± 1.17%) or curcumin (47.81 ± 1.12%) in D-gal and EGCG (65.62 ± 1.60%) or curcumin (54.79 ± 1.55%) in NA, much higher prevention from the dropping trend of FR was determined in the combined EGCG and curcumin in D-gal (70.93 ± 1.33%; F_1,14_ = 0.17, p < 0.001; F_1,14_ = 0.38, p < 0.001, respectively; Fig. 4) and NA (70.20 ± 1.61%; F_1,14_ = 0, p < 0.01; F_1,14_ = 0.07, p < 0.0001, respectively; Fig. 5) groups (One-Way ANOVA followed by post hoc Tukey’s test). The FR distinguished in the co-administered EGCG and curcumin were comparable to the FR exhibited by Ast + D-gal (67.22 ± 1.28%; p > 0.05; Fig. 4) and Ast + NA (65.13 ± 2.92%; p > 0.05; Fig. 5) groups.

A similar trend of freezing response was exhibited in control, aging, and treatment models on day 31b (After performing day 31a of context) in the presence of CS application during the last 3 min of the cued test. In contrast to the monotherapy of EGCG (51.66 ± 1.33%; Fig. 4) or curcumin (41.25 ± 1.25%; Fig. 4) in D-gal and EGCG (59.16 ± 0.99%; Fig. 5) or curcumin (49.16 ± 0.62%; Fig. 5) in NA, there was a statistically substantial change in FR distinguished in the combined EGCG and curcumin in D-gal (62.08 ± 1.77%; F_1,14_ = 0.96, p < 0.01; F_1,14_ = 1.15, p < 0.0001, respectively; Fig. 4) and NA (67.29 ± 1.96%; F_1,14_ = 7.57, p < 0.01; F_1,14_ = 13.51, p < 0.0001, respectively; Fig. 5) groups (One-Way ANOVA followed by post hoc Tukey’s test). The FR detected by the combination treatment of EGCG and curcumin was comparable to the Ast + D-gal (57.84 ± 2.10%; p > 0.05; Fig. 4) and Ast + NA group mice (63.26 ± 2.69%; p > 0.05; Fig. 5).

### Effect of EGCG + curcumin on oxidative overload biomarkers

#### GSH, SOD, and CAT

**Figure 6:**
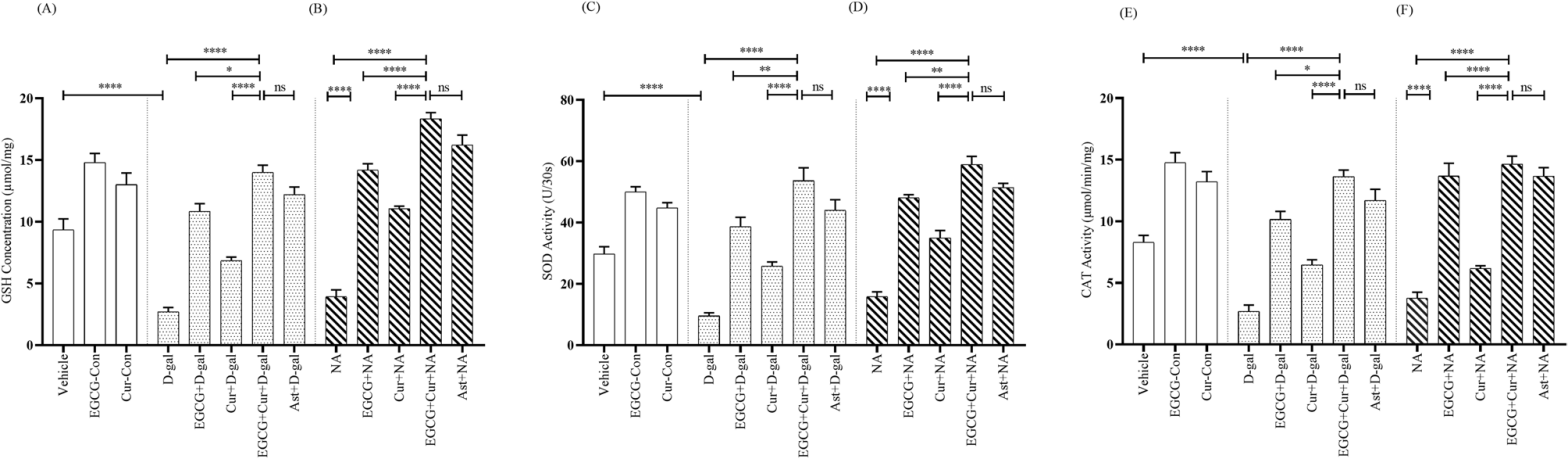
Effect of EGCG + Curcumin on GSH concentration (**A**,**B**), SOD (**C**,**D**) and CAT (**E**,**F**) activity in D-gal and NA mice group. The level of GSH, SOD and CAT activity were detected by using bioassay technique among young adult (vehicle, EGCG-Con, Cur-Con), drug induced aging (D-gal, EGCG + D-gal, Cur + D-gal, EGCG + Cur + D-gal, and Ast + D-gal), and nature induced aging (NA, EGCG + NA, Cur + NA, EGCG + Cur + NA, and Ast + NA) groups. GSH level, SOD and CAT activity were expressed in μmol/mg, U/30 s, and μmol/min/mg, respectively. Data was presented as mean ± SEM, n = 8 each group; *p < 0.05, **p < 0.01 ***p < 0.001, ****p < 0.0001, *ns* not significant.

Two-way ANOVA followed by post hoc Tukey’s test across the aging (Young, D-gal, and NA) and treatment (EGCG, Cur, EGCG + Cur, Ast, and no treatment) demonstrated a significant effect of the aging on GSH (F_2,91_ = 82.03, p < 0.0001; Fig. 6A,B), SOD (F_2,91_ = 41.77, p < 0.0001; Fig. 6C,D) and CAT (F_2,91_ = 60.26, p < 0.0001; Fig. 6E,F), treatment on GSH (F_4,91_ = 139.93, p < 0.0001), SOD (F_4,91_ = 112.49, p < 0.0001) and CAT (F_4,91_ = 106.31, p < 0.0001) and a significant interaction between aging and treatment on GSH (F_6,91_ = 3.46, p < 0.001), CAT (F_6,91_ = 3.50, p < 0.001), and SOD (F_6,91_ = 1.38, p < 0.05). The level of GSH declined drastically in the D-gal (2.71 ± 0.33 μmol/mg; Fig. 6A) and NA (3.92 ± 0.54 μmol/mg; Fig. 6B) groups, denoting a statistically remarkable difference compared with the Vehicle (p < 0.0001), EGCG-Con (p < 0.0001) and Cur-Con (p < 0.0001) groups (Fig. 6A). Contrarily, in comparison to the discrete therapy of EGCG (10.84 ± 0.61 μmol/mg; Fig. 6A) or curcumin (6.83 ± 0.30 μmol/mg; Fig. 6A) in D-gal and EGCG (14.17 ± 0.52 μmol/mg; Fig. 6B) or curcumin (11.06 ± 0.18 μmol/mg; Fig. 6B) in NA, much higher protection from the dropping tendency of GSH level was detected in the combined EGCG and curcumin in D-gal (14 ± 0.57 μmol/mg; F_1,14_ = 0, p < 0.05; F_1,14_ = 0.56, p < 0.0001; respectively; Fig. 6A) and NA (18.32 ± 0.51 μmol/mg; F_1,14_ = 0.94, p < 0.0001; F_1,14_ = 2.04, p < 0.0001, respectively; Fig. 6B) mice (One-Way ANOVA followed by post hoc Tukey’s test). The protection from the descending trend of GSH by the combined EGCG and curcumin in D-gal and NA mice were equivalent to the Ast + D-gal (12.20 ± 0.60 μmol/mg; p > 0.05; Fig. 6A) and Ast + NA (16.20 ± 0.81 μmol/mg; p > 0.05; Fig. 6B).

Similar to GSH, the level of SOD activity reduced significantly in D-gal (9.51 ± 1.05 U/30 s; Fig. 6C) and NA (15.83 ± 1.58 U/30 s; Fig. 6D) compared to Vehicle (p < 0.0001), EGCG-Con (p < 0.0001) and Cur-Con (p < 0.0001) groups (Fig. 6C,D). Contrarily, contrasting to the monotherapy of EGCG (38.67 ± 3.02 U/30 s; Fig. 6C) or curcumin (25.80 ± 1.33 U/30 s; Fig. 6C) in D-gal and EGCG (48.07 ± 1.01 U/30 s; Fig. 6D) or curcumin (34.94 ± 2.46 U/30 s; Fig. 6D) in NA, the co-administered treatment of EGCG and curcumin demonstrated higher prevention from the dropping trend of SOD activity in D-gal (53.6 ± 4.25 U/30 s; F_1,14_ = 0.22, p < 0.01, F_1,14_ = 1.95, p < 0.0001, respectively; Fig. 6C) and NA (58.89 ± 2.70 U/30 s; F_1,14_ = 5.85, p < 0.01, F_1,14_ = 0.27, p < 0.0001, respectively; Fig. 6D) mice (A One-Way ANOVA followed by post hoc Tukey’s test). This protection was comparable to the Ast + D-gal (43.99 ± 3.44 U/30 s; p > 0.05; Fig. 6C) and Ast + NA (51.45 ± 1.30 U/30 s; p > 0.05; Fig. 6D).

Similar to the GSH and SOD activity, the control, aging, and treatment models revealed the same pattern of effect in CAT activity. In comparison to the monotherapy of EGCG (10.15 ± 0.64 μmol/min/mg; Fig. 6E) or curcumin (6.46 ± 0.40 μmol/min/mg; Fig. 6E) in D-gal and EGCG (13.67 ± 1.04 μmol/min/mg; Fig. 6F) or curcumin (6.16 ± 0.21 μmol/min/mg; Fig. 6F) in NA, much higher prevention from the descending trend of the activity of CAT was observed in the combined EGCG and curcumin in D-gal (13.62 ± 0.52 μmol/min/mg; F_1,14_ = 0.92, p < 0.05; F_1,14_ = 0.10, p < 0.0001, respectively; Fig. 6E) and NA (14.63 ± 0.65 μmol/min/mg; F_1,14_ = 1.63, p < 0.0001; F_1,14_ = 5.28, p < 0.0001, respectively; Fig. 6F) mice (One-Way ANOVA followed by post hoc Tukey’s test). The protection from the declining trend of CAT activity by the combined EGCG and curcumin therapy was equivalent to the Ast + D-gal (11.68 ± 0.90 μmol/min/mg; p > 0.05; Fig. 6E) and Ast + NA (13.66 ± 0.68 μmol/min/mg; p > 0.05; Fig. 6F) groups.

#### AOPP, NO, and MDA

**Figure 7:**
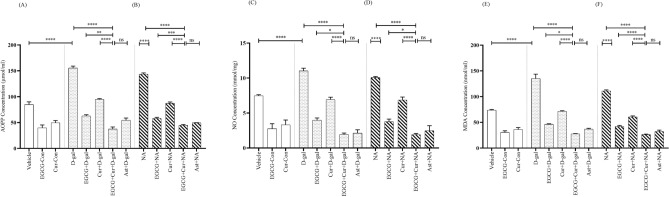
Effect of EGCG + Curcumin on AOPP (**A**,**B**), NO (**C**,**D**), and MDA (**E**,**F**) concentration in D-gal and NA mice group. The AOPP, NO, and MDA level were assessed by using bioassay technique among young adult (vehicle, EGCG-Con, Cur-Con), drug induced aging (D-gal, EGCG + D-gal, Cur + D-gal, EGCG + Cur + D-gal, and Ast + D-gal), and nature induced aging (NA, EGCG + NA, Cur + NA, EGCG + Cur + NA, and Ast + NA) groups. AOPP, NO, and MDA level were represented in μmol/ml, mmol/mg, and nmol/ml, respectively. Data was presented as mean ± SEM, n = 8 each group; *p < 0.05, **p < 0.01, ****p < 0.0001, *ns* not significant.

A statistical comparison across the aging (Young, D-gal, and NA) and treatment (EGCG, Cur, EGCG + Cur, Ast, and no treatment), using a two-way ANOVA followed by post hoc Tukey’s test, detected a substantial effect of the aging on AOPP (F_2,91_ = 155.58, p < 0.0001; Fig. 7A,B), NO (F_2,91_ = 34.12, p < 0.0001; Fig. 7C,D) and MDA (F_2,91_ = 111.80, p < 0.0001; Fig. 7E,F), treatment on AOPP (F_4,91_ = 339.53, p < 0.0001), NO (F_4,91_ = 146.53, p < 0.0001) and MDA (F_4,91_ = 331.88, p < 0.0001) and a significant interaction between aging and treatment on AOPP (F_6,91_ = 10.80, p < 0.0001), NO (F_6,91_ = 2.29, p < 0.05) and MDA (F_6,91_ = 10.43, p < 0.0001).

The AOPP level was sharply elevated to 155.27 ± 3.79 μmol/ml in D-gal (Fig. 7A) and 143.57 ± 2.35 μmol/ml in NA (Fig. 7B) groups compared to Vehicle (p < 0.0001), EGCG-Con (p < 0.0001), and Cur-Con (p < 0.0001) (Fig. 7A). Contrarily, in contrast to the discretely administered EGCG (62.73 ± 2.43 μmol/ml; Fig. 7A) or curcumin (94.63 ± 1.65 μmol/ml; Fig. 7A) in D-gal and EGCG (58.01 ± 2.03 μmol/ml: Fig. 7B) or curcumin (87.25 ± 2.49 μmol/ml; Fig. 7B) in NA, much higher prevention from the elevating tendency of the AOPP level was detected in the combined EGCG and curcumin therapy in D-gal (37.54 ± 3.98 μmol/ml; F_1,14_ = 6.71, p < 0.01; F_1,14_ = 18.98, p < 0.0001, respectively; Fig. 7A) and NA (44.74 ± 1.41 μmol/ml; F_1,14_ = 3.44, p < 0.001; F_1,14_ = 0.16, p < 0.0001, respectively; Fig. 7B) groups (One-Way ANOVA, post hoc Tukey’s test). This prevention from the rising tendency of AOPP level was equivalent to the Ast + D-gal (54.37 ± 4.34 μmol/ml; p > 0.05; Fig. 7A) and Ast + NA (49.47 ± 0.28 μmol/ml; p > 0.05; Fig. 7B) groups.

Similar to AOPP level, the NO level was greatly elevated in D-gal (11.02 ± 0.38 mmol/mg; Fig. 7C) and NA (10.07 ± 0.13 mmol/mg, Fig. 7D) compared to Vehicle (p < 0.0001), EGCG-Con (p < 0.0001) and Cur-Con (p < 0.0001) groups (Fig. 7C). In comparison to the monotherapy of EGCG (3.97 ± 0.31 mmol/mg; Fig. 7C) or curcumin (6.91 ± 0.34 mmol/mg; Fig. 7C) in D-gal and EGCG (3.75 ± 0.38 mmol/mg; Fig. 7D) or curcumin (6.84 ± 0.44 mmol/mg) in NA, much higher prevention from the elevating trend of NO was determined in the combined EGCG and curcumin in D-gal (1.93 ± 0.19 mmol/mg; F_1,14_ = 2.10, p < 0.05; F_1,14_ = 0.61, p < 0.0001, respectively; Fig. 7C) and NA (1.91 ± 0.15 mmol/mg; F_1,14_ = 2.06, p < 0.05; F_1,14_ = 1.40, p < 0.0001, respectively; Fig. 7D) groups, detected by One-Way ANOVA followed by post hoc Tukey’s test. This protection from the escalating trend of NO by the combined EGCG and curcumin was comparable to the Ast + D-gal (2.12 ± 0.48 mmol/mg; p > 0.05; Fig. 7C) and Ast + NA (2.46 ± 0.72 mmol/mg; p > 0.05; Fig. 7D) groups.

Similar to the AOPP and NO level, the control, aging and treatment models demonstrated a similar pattern of effect in MDA Level. In contrast to the monotherapy of EGCG (46.31 ± 0.93 nmol/ml; Fig. 7E) or curcumin (71.50 ± 1.14 nmol/ml; Fig. 7E) in D-gal and EGCG (42.11 ± 1.87 nmol/ml; Fig. 7F) or curcumin (60.60 ± 1.88 nmol/ml; Fig. 7F) in NA, much higher protection from the escalating tendency of MDA was distinguished in the combined EGCG and curcumin therapy in D-gal (27.64 ± 0.61 nmol/ml; F_1,14_ = 2.19, p < 0.05; F_1,14_ = 7.18, p < 0.0001, respectively; Fig. 7E) and NA (26.46 ± 0.79 nmol/ml; F_1,14_ = 4.66, p < 0.0001; F_1,14_ = 11.32, p < 0.0001, respectively; Fig. 7F). This prevention from the elevating trend of MDA level by the combined EGCG and curcumin was comparable to the Ast + D-gal (36.77 ± 1.70 nmol/ml; p > 0.05; Fig. 7E) and Ast + NA (32.22 ± 2.72 nmol/ml; p > 0.05; Fig. 7F).

## Discussion

Previously we assessed the effect of monotherapy to treat memory dysfunction in D-gal and NA mice, induced by oxidative stress pathway^15^. Contrarily, it is established that interactive systems of molecules, cells, and multiple pathways contribute to the process of aging^26^. Additionally, considering the intricacy of the process of aging, it can be stated that combined therapy may bring a successful treatment strategy by producing substantial protection from memory impairment in the aging population^28^. Therefore, the previous study certainly missed to find out a successful treatment approach by assessing the effect of single therapy on diverse pathology of aging-induced memory impairment. In this current study, we assessed the effects of combination therapy to treat memory impairment in D-gal and NA mice. We found that the combined therapy of EGCG and curcumin exhibits a more substantial improvement of retention and fear memory by ameliorating oxidative stress biomarkers in contrast to their monotherapy in drug and nature-induced aging animals.

Similar to our previous study^15^, the D-gal administered, and NA animals displayed less retention time (RT) and freezing response (FR) in the PA (Fig. 1) and CFC (Figs. 2, 3, 4, 5) tasks, respectively. Studies showed that a large quantity of D-gal generated excessive reactive oxygen species (ROS)^10^ by impairing redox homeostasis^12^, commonly observed in the natural progression of aging mice^15^. This high production of ROS induces brain-derived neurotrophic factor (BDNF) dysregulation neuroinflammation, and cellular apoptosis, contributing to memory dysfunction^29^. Another study revealed that D-gal caused memory and learning deficit by reducing adult neurogenesis^30^ and synaptic protein expressions in the hippocampus^31^, a vital region processing learning, and memory in behavioral tasks^32,33^. Apart from the involvement of hippocampus in memory, a decreased functional connectivity between amygdala and hippocampus and an increased connectivity between amygdala and dorsolateral prefrontal cortex observed in older adults in learning emotion-based memory^34^. Our findings from behavioral studies agree with the previous study^15^, suggesting, D-gal induces natural aging and impairs learning and memory in mice.

The combined EGCG and curcumin therapy in both D-gal and naturally induced aging mice groups showed powerful protection of retention time and freezing response in PA and CFC tasks, respectively (Figs. 1, 2, 3, 4, 5), suggesting better improvement of learning and memory compared to the monotherapy of EGCG or curcumin in both mouse models. Evidence showed that EGCG reversed D-gal administered memory impairment in aging mice^14^. A study demonstrated that EGCG improved cognitive deficits by producing neuroprotective, anti-inflammatory, and molecular effects in mice^35^. EGCG exerts neuroprotective effects by substantiating the functions of antioxidant enzymes and inhibiting the damage of advanced glycation end product in aged animals^14^. It was also evident that EGCG exhibits anti-inflammatory actions by inhibiting the expression of inducible NO synthase (iNOS) and reducing nuclear factor kappa B (NF-κB) and activator protein-1 activities (AP-1)^36^. Moreover, EGCG improved cognitive dysfunction by suppressing the activity of b-secretase^37^, causation of β-amyloid in APP695 expressing neurons^38^, formation of Ab fibrils^39^, and caspase activity in neuronal cells of hippocmapus^40^.

Curcumin produces neuroprotective effects by exerting antioxidant, anti-aging, and anti-neuroinflammation properties^41,42^. A study showed that curcumin protects memory from impairment in D-gal and NA-induced memory impairment^15^ by regulating degeneration, proliferation, and senescence of neuronal cells^43^. Additionally, curcumin was found to improve cognitive dysfunction by increasing synaptic density in the AD animal^44^. Apart from these effects, curcumin increased the permeability and bioavailability of EGCG^45^ and produces synergistic effects^46^ in animals. Therefore, our present study concludes that the combined EGCG and curcumin substantiate the powerful protection via producing potential pharmacological actions in brain aging.

Similar to the findings of the previous study^15^, we found a sharply decreased activity of antioxidants including GSH (Fig. 6A,B), SOD (Fig. 6C,D) CAT (Fig. 6E,F) in D-gal treated and NA animals. Moreover, an equivalent level of antioxidant activity was found in both classified mouse models (Fig. 6). GSH reduces H_2_O_2_ to form H_2_O by interacting with ROS and RNS directly^47^. Similarly, superoxide dismutase (SOD) accelerates the disproportionation of O_2_^·−^ into H_2_O_2_ and O_2_^48^. In addition, Catalase (CAT) converts H_2_O_2_ into H_2_O and O_2_^48^. Consequently, the cell stays protected from the damaging effects of H_2_O_2_. These non-enzymatic and enzymatic antioxidant molecules (GSH, SOD, CAT) avert oxidation in the plasma membrane and modulate redox homeostasis^49^. Contrarily, a substantial decline of GSH, SOD, and CAT molecules fails to prevent the uncontrolled production of species containing reactive oxygen (ROS) and nitrogen (RNS)^50^ induced by D-gal^51^ and NA^52^. On the other hand with the low level of antioxidants, we detected a high level of AOPP (8A and 8B), NO (8C and 8D), and MDA (8E and 8F) in D-gal treated and NA mice, suggesting the emergence of oxidative stress in mice^53^. A high level of oxidized protein signals generated an excess amount of AOPP^53^. A high amount of NO induces more RNS, such as ^·^NO_2_ and N_2_O_3_^54^. Similarly, an increased level of MDA induces more ROS^55^. Therefore, a large amount of these reactive species (MDA, AOPP, and NO) mediate oxidative stress in aging mice^15^.

In the current study, we noted that combined EGCG and curcumin-treated mice showed powerful protection against the decreased activity of GSH, SOD, CAT (Fig. 6), and increasing level of AOPP, NO, and MDA (Fig. 7) in the brain compared to the monotherapy of EGCG or curcumin. Moreover, combined EGCG and curcumin were comparable to Ast, a standard antioxidant, in modulating the antioxidant level. A study showed that EGCG improved memory dysfunction by elevating the GSH and SOD activity^56^ in D-gal-induced aging mice^14^. Another study showed that EGCG decreased hippocampal MDA^56^ and cortico-hippocampal ROS and improved memory in AD mice model^57^. These antioxidant activities of EGCG are modulated by binding with several aging and oxidative stress-associated proteins such as KEAP1^58^, BACE1^59^, protein kinase C^60^, p53, Bax, Bcl-XL, and COX^61^. A previous study showed that curcumin improved memory by modulating the level of GSH, SOD, CAT, AOPP, NO, and MDA in aging mice^15^, by interacting with several proteins, such as kelch-like ECH-associated protein 1 (KEAP1), amine oxidase [flavin-containing] A (MAOA), beta-secretase 1 (BACE1), glutathione S-transferase omega-1 (GSTO1), and glutathione S-transferase A1 (GSTA1)^15^. Together previous and present findings suggested that the combined therapy of EGCG and curcumin substantiate greater protection against oxidative stress injury by regulating the oxidative biomarkers and their associated proteins in aging.

## Conclusion

We investigated the beneficial effects of EGCG and curcumin on oxidative stress in the two robust aging mice models by performing behavioral and biochemical studies. A combination of EGCG with curcumin exhibits greater protection from aging-related memory impairment by modulating oxidative stress biomarkers. To further substantiate the findings, molecular and cellular studies need to be conducted to examine the regulatory effect of combined EGCG and curcumin on oxidative biomarkers in aging-induced learning impairment.

## Materials and methods

### Chemicals and reagents

We acquired thiobarbituric acid (TBA, T5500), epigallocatechin 3 gallate (EGCG, PHL89656), astaxanthin (Ast, SML0982), curcumin (C1386), trichloroacetic acid (TCA, T6399), D galactose (D-gal, G0750) from Sigma-Aldrich (Germany), and protease inhibitor cocktail from Sigma, Saint Louis, MO, USA. We used all required materials, chemicals, and reagents of the highest standard for analytical applications in this study.

### Animals

We chose male mice to generate a stable reading in fear-based tasks since a previous study showed that female mice may bring unexpected memory deficits owing to their increasing anxious behavioral endophenotypes modulated by age^62^. We selected *Swiss albino* strain because of its several advantages including strong learning interest, intricate intelligence in memory paradigms and decreasing variability of experiments in aging study^63^. However, this strain may inherit a rare genetic disorder, albinism, that impairs visual spatial attention^64^ required for passive avoidance^65^ and contextual fear conditioning^66^. Therefore, we performed visual placing response, demonstrated by W. M. Fox in previous protoco^67^, to assess the visual system before selecting our experimental mice. Briefly, mouse was lowered by suspending its tail toward a metal object while we ensured no contact of vibrissae with the metal object. Mouse, which showed a clear placing reaction by raising its head and extending forelimbs on the metal object, was selected in this study. One hundred four healthy *Swiss albino* mice were chosen and separated into three age groups, Group—1 (Young adult; 24 mice): 6–8 weeks old, with an average weight of 25 gm; Group—2 (Drug induced aging; 40 mice): 6–8 weeks old, treated with D-gal; and Group—3 (Nature induced aging; 40 mice): 10–12 months of age with an average weight of 40 gm, did not receive D-gal. Eight mice comprised each subgroup of age category. Each mouse was fed ad-libitum. Mice were housed in a 12:12 h light–dark cycle while maintaining 55% ± 15% relative humidity at 25 °C temperature.

### Drugs preparation and experimental design

EGCG solution at 0.2 mg/ml concentration was prepared by dissolving it into distilled water^68^. Curcumin was administered at 1 ml per 100 g body weight after suspending in a percentage of 0.25 w/v sodium carboxymethylcellulose^69^. The D-gal solution used to induce aging was prepared before each session of administration by dissolving it into 0.9% of saline^70^. Ast solution at 20 mg/20 ml used as a standard agent to compare the antioxidant effect of combined EGCG and curcumin was prepared by dissolving it into distilled water^71^. We treated and divided one hundred four mice into following subgroups of each age category and assessed the mice using multiple apparatus at the same time by dedicated expert experimenters.

#### Young adult groups

1. Vehicle (n = 8): each mouse was administered sodium carboxymethylcellulose at 0.25% w/v orally^69^ once daily. This vehicle group was considered for both normal and drug-induced aging mouse models.
2. EGCG—Control (Positive Control; n = 8): each mouse was administered EGCG at 2 mg/kg^68^ orally once regularly.
3. Curcumin-Control (Positive Control; n = 8): each mouse was administered curcumin at 30 mg/kg^15,69^ orally once daily.

#### Drug induced aging groups

1. D-gal (n = 8): each mouse was administered D-gal at 100 mg/kg intraperitoneally^15,70^ once daily.
2. EGCG + D-gal (n = 8): each mouse was administered EGCG at 2 mg/kg^68^ orally and D-gal at 100 mg/kg^15,70^ intraperitoneally once daily.
3. Curcumin + D-gal (n = 8): each mouse was administered curcumin at 30 mg/kg^15,69^ orally and D-gal at 100 mg/kg^15,70^ intraperitoneally once daily.
4. EGCG + Curcumin + D-gal (n = 8): each mouse was administered EGCG at 2 mg/kg^68^, curcumin at 30 mg/kg, orally^15,69^, and D-gal at 100 mg/kg intraperitoneally^15,70^ once daily.
5. Ast (standard antioxidant) + D-gal (n = 8): each mouse was administered Ast at 20 mg/kg^15,53^ orally and D-gal at 100 mg/kg^15,70^ intraperitoneally once daily.

#### Nature induced aging groups

1. NA (n = 8): each mouse had adequate accession to pellets and water regularly.
2. EGCG + NA (n = 8): each mouse was administered EGCG at 2 mg/kg^68^ orally on regular basis.
3. Curcumin + NA (n = 8): each mouse was administered curcumin at 30 mg/kg orally^15,69^ once on daily basis.
4. EGCG + Curcumin + NA (n = 8): each mouse was administered EGCG at 2 mg/kg^68^ and curcumin at 30 mg/kg^15,69^, orally, once daily.
5. Ast + NA (n = 8): each mouse was administered Ast at 20 mg/kg orally^15,53^ once daily.

**Figure 8:**
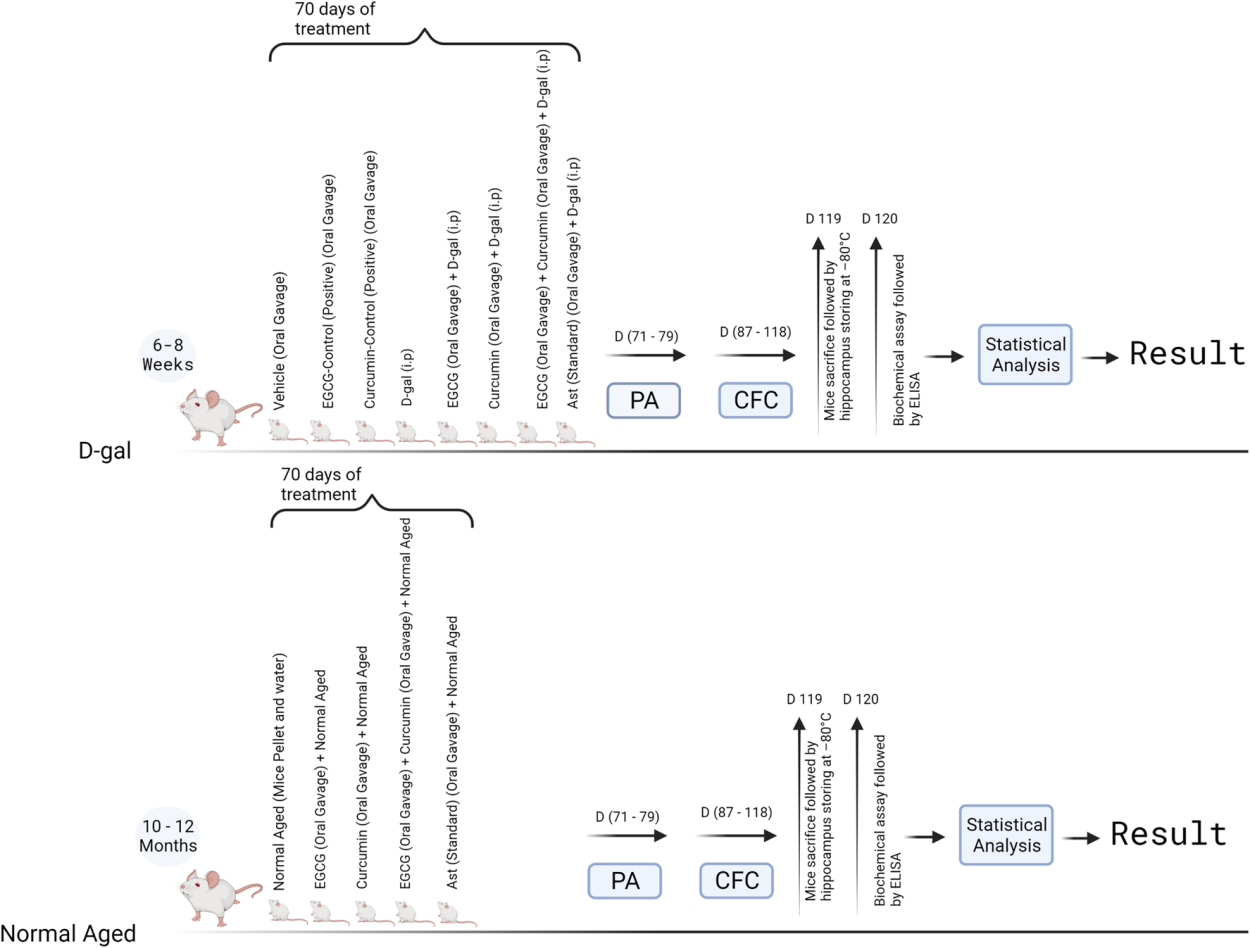
Schematic presentation of whole experimental procedure: the treatment continued for ten weeks. After that, the behavioral parameters were detected using passive avoidance (PA) and contextual fear conditioning (CFC). The biomarkers were measured after completing the behavioral tasks.

After continuing the treatment for ten weeks, we performed PA and CFC tasks to assess the retention and fear memory, respectively. Finally, we euthanized all mice to collect brain samples to detect oxidative markers. The whole process of this research study is illustrated in Fig. 8.

### Passive avoidance and contextual fear conditioning test

The PA test was performed as stated in the protocol of Tabrizian^72^. The whole procedure and constitution of the PA chamber are demonstrated in our previous study^15^. Briefly, the training session consisted of five consecutive days (2–6) from the next day of the habituation session which took place on day 1 to make mice familiar with the interior environment of the experimental apparatus. Finally, after 24 and 48 h of the completion of the whole training session, a stopwatch was used to capture the retention time.

After a week of performing the PA test, we conducted the CFC test by following the protocol of Shoji^73^ and our previous study^15^. Shortly, in the conditioning session (Day 1 of CFC), each mouse was carefully housed in the transparent acrylic compartment and allowed to acclimate to the experimental instruments for 2 min. Next, the mice received a conditioned stimulus (CS) of 55 dB white noise for 30 s co-terminated with an unconditioned stimulus (US) of 0.3 mA foot shock for 2 s. This pairing of CS-US was presented two times more while maintaining an inter-stimulus interval of 2 min. On day 2a (after 24 h) and 31a (after 30 days) of the conditioning session, the context test was performed in the same chamber. A few hours later after completing the context test, the cued test was conducted in the triangle-shaped compartment (33 × 29 × 32 cm) on day 2b (after the completion of context test of day 2a) and day 31b (after the completion of context test of day 31a). The freezing response of mice was detected by using an infrared video system connected to a computer (Med Associates, Inc. USA)^74^.

### Tissue processing and oxidative stress measurement

The biochemical analysis was performed on mice of each group. Like in our previous study^15^, mice were sacrificed by decapitation followed by perfusion with 0.9% NaCl after anesthetizing by ketamine at 50 mg/ml, purchased from Renata Ltd., Bangladesh. The hippocampal tissue was instantaneously preserved at − 80 °C after microdissection. Ultra-Turrax T25, manufactured from the USA, was performed to prepare hippocampal homogenate (10%; w/v) by using the mixture of buffer sodium phosphate (1 × PBS; pH 7.0) and 1:100 protease inhibitor. An ultrasonic processor was used to sonicate the homogenized tissue at a 5-s cycle for 150 s followed by centrifugation at 10,000 rpm (RCF 11200) at 4 °C for 10 min. After that, 0.1× PBS buffer was used to dilute the clear supernatants. Finally, the biochemical analysis was performed after collecting the clear supernatants. Total protein concentration was detected by the method of Lowry^75^. The level of GSH was measured by following the previous protocol^15,76,77^. 1 ml of homogenized hippocampal tissue was mixed with phosphate buffer (2.7 ml; 0.1 M; pH 8) and 5,5-dithiol-bis (0.2 ml). The progression of color was determined immediately at 412 nm and the data was illustrated in μmol/mg protein. The SOD level was measured similarly to the modified previous method^15,78,79^. The reaction mixture was composed of methionine (13 mM), riboflavin (2 mM), sodium phosphate (50 mM; pH 7.8), EDTA (100 mM), nitroblue tetrazolium (NBT, 75 mM), and homogenized hippocampal tissue (2 ml). After the manifestation of blue formazan at 560 nm, the change in absorbance was recorded for each sample and the data was represented in U/30 s. The CAT activity was estimated spectrometrically by following the previous method^15,80^. A 1.5 ml reaction mixture consisted of phosphate buffer (1.0 ml; 0.01 M; pH 7.0), homogenized hippocampal tissue (0.1 ml), and H_2_O_2_ (0.4 ml of 2 M). After the addition of dichromate-acetic acid reagent (2.0 ml), the mixture (1:3) of potassium dichromate, and glacial acetic acid, the reaction stopped, and the data were expressed in μmol/min/mg protein. AOPP was determined spectrophotometrically by adopting the previous method^15,81,82^. The phosphate-buffered saline (PBS) was used to dilute homogenized hippocampal tissue (50 μl) at a ratio of 1:2. The calibration curve was prepared by using Chloramine T (0–100 mmol/l). Each well was poured with acetic acid (50 μl) and potassium iodide (100 μl; 1.16 M) while PBS was used as a blank. Finally, the absorbance at 340 nm was detected and the data was represented in chloramine units (μmol/ml). The level of NO was detected, using the Griess-Illosvoy reagent, based on the previous method^15,83^. Naphthyl ethylene diamine dihydrochloride (0.1% w/v) was used as a substitute for 1-napthylamine (5%) for modifying Griess–Illosvoy reagent. The PBS was used to dilute NED (1 ml), sulfanilamide (1 ml), phosphate buffer saline (0.5 ml), and homogenized hippocampal tissue at the ratio of 2:8. The incubation was done in a 96-well plate at 25 °C for 15 min^76^. Finally, the absorbances were detected in the spectrophotometer at 540 nm against the blank readings and the data was represented in mmol/mg. The level of MDA was detected using colorimetric analysis while determining TBARS based on the previous protocol^15,84^. The Tris–HCl buffer (pH 7.5) containing homogenized hippocampal tissue (0.1 ml) was treated with 2 ml of TBA (0.37%)-TCA (15%)-HCl (0.25 N) reagent (1:1:1 ratio), later kept in a water bath for 15 min at 70 °C and cooled. The absorbance was determined at 535 nm against the reference blank^85^ and finally, the MDA level (nmol/ml) was measured based on a standard curve.

### Data analysis

The GraphPad Prism (version 8.0.1 for windows, GraphPad software, www.graphpad.com) and SPSS software (IBM SPSS 29) was used to execute all analyses. A One-Way ANOVA followed by post hoc Tukey’s test detected the effects of groups and a two-Way ANOVA followed by post hoc Tukey’s test determined the main effects of aging, compounds, and their interactions. Data presented as mean ± SEM (standard error of the mean) and the difference was considered significant when the p value was less than 0.05.

### Ethical approval

The present study protocol was approved by the institutional animal care and use committee (IACUC) of North South University (2020/OR-NSU/IACUC-No.0903, SL no39). The experimental procedures were maintained and executed at the pathogen-free facility according to the NIH Guide for the care and use of laboratory animals. Maximum efforts were given to reduce animal quantity and ensure their comfort. This study is reported in accordance with ARRIVE guidelines (Animal Research: Reporting of In Vivo Experiments).

## Limitation

At present we assessed the beneficial effects of EGCG and curcumin on several oxidative biomarkers in one strain of aging animals (male mice only) by performing PA and CFC studies. To further understand the outcomes of aging and the treatment therapies, considering other strains of mice of both male and female, measuring some functional outcomes of increased oxidative stress, such as DNA damage and/or apoptosis and strengthening the validity of behavioral studies by using other reward-based tests, such as radial 8-arm maze or the Hebb-Williams maze, and tests require movement, such as the Barnes maze or Morris water maze need to be conducted.

### Supplementary Information

Supplementary Figures.

Supplementary Legends.

## Data Availability

The data supporting the present study findings are obtainable from corresponding authors upon reasonable request.
