# Supplementary figures and images for: Combination of epigallocatechin 3 gallate and curcumin improves d-galactose and normal-aging associated memory impairment in mice
Source: Sci Rep. 2023 Aug 4;13:12681. doi: 10.1038/s41598-023-39919-4 (PMC10403524; doi:10.1038/s41598-023-39919-4)

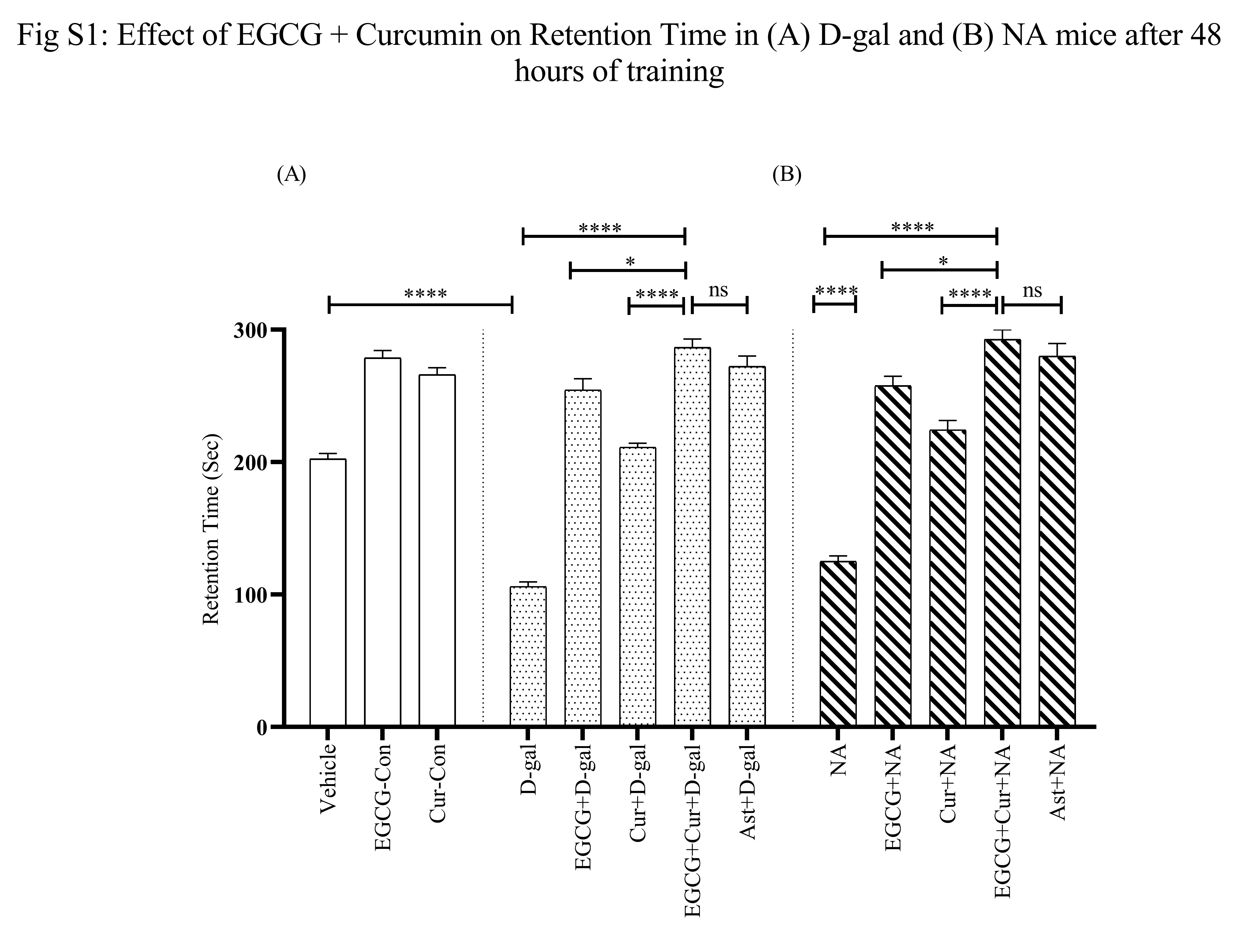

Supplement: Supplementary file 1 — Supplementary Figures. [file 41598_2023_39919_MOESM1_ESM.tif]
