# Supplementary material for: Combination of epigallocatechin 3 gallate and curcumin improves d-galactose and normal-aging associated memory impairment in mice
Source: Sci Rep. 2023 Aug 4;13:12681. doi: 10.1038/s41598-023-39919-4 (PMC10403524; doi:10.1038/s41598-023-39919-4)
Supplement: Supplementary file 2 — Supplementary Legends. [file 41598_2023_39919_MOESM2_ESM.docx]

**Fig S1**. Effect of EGCG + Curcumin on RT in D-gal (A) and NA (B) mice group after 48 hours of training. The RT was detected by performing PA tasks among young adult (Vehicle, EGCG-Con, Cur-Con), drug induced aging (D-gal, EGCG + D-gal, Cur + D-gal, EGCG + Cur + D-gal, and Ast + D-gal), and nature induced aging (NA, EGCG + NA, Cur + NA, EGCG + Cur + NA, and Ast + NA). RT was illustrated in second. Data was presented as mean ± SEM, n=8 each group; ****p < 0.0001, ns = not significant.
